# Heterogeneous Mobile Phone Ownership and Usage Patterns in Kenya

**DOI:** 10.1371/journal.pone.0035319

**Published:** 2012-04-25

**Authors:** Amy Wesolowski, Nathan Eagle, Abdisalan M. Noor, Robert W. Snow, Caroline O. Buckee

**Affiliations:** 1 Department of Engineering and Public Policy, Carnegie Mellon University, Pittsburgh, Pennsylvania, United States of America; 2 Department of Epidemiology, Harvard School of Public Health, Boston, Massachusetts, United States of America; 3 Malaria Public Health and Epidemiology Group, Centre of Geographic Medicine, KEMRI-Wellcome Trust-University of Oxford Collaborative Programme, Nairobi, Kenya; 4 Centre for Tropical Medicine, Nuffield Department of Clinical Medicine, University of Oxford, Oxford, United Kingdom; 5 Center for Communicable Disease Dynamics, Harvard School of Public Health, Boston, Massachusetts, United States of America; Universitat Rovira i Virgili, Spain

## Abstract

The rapid adoption of mobile phone technologies in Africa is offering exciting opportunities for engaging with high-risk populations through mHealth programs, and the vast volumes of behavioral data being generated as people use their phones provide valuable data about human behavioral dynamics in these regions. Taking advantage of these opportunities requires an understanding of the penetration of mobile phones and phone usage patterns across the continent, but very little is known about the social and geographical heterogeneities in mobile phone ownership among African populations. Here, we analyze a survey of mobile phone ownership and usage across Kenya in 2009 and show that distinct regional, gender-related, and socioeconomic variations exist, with particularly low ownership among rural communities and poor people. We also examine patterns of phone sharing and highlight the contrasting relationships between ownership and sharing in different parts of the country. This heterogeneous penetration of mobile phones has important implications for the use of mobile technologies as a source of population data and as a public health tool in sub-Saharan Africa.

## Introduction

As the adoption of mobile phones continues to rise rapidly so do the opportunities to directly engage with populations for policy purposes, as well as to study their dynamics on a scale previously impossible. The diffusion of mobile phone technologies has been particularly striking in Africa, home to over 400 million mobile phone subscribers [1]. The unexpected prevalence of mobile devices in poor, rural populations has raised the possibility of using “mHealth” approaches to provide public health services directly to communities that have traditionally been hard to reach [2]. Furthermore, the data passively generated each time a person uses their mobile phone to call and text can be used to understand large-scale patterns of individual behaviors like mobility and communication [3], [4], [5], [6]. Studies of this kind have highlighted the consistency of travel patterns in high-income countries and shown how wealth relates to social network structure [3], [4]. To date there have been almost no analyses of the dynamics of populations in low-income countries, however. A prerequisite to studies of this kind and to the effective use of mHealth strategies is an understanding of the distribution of mobile phones within populations, and the ways in which people use their phones in different communities. Surprisingly, however, the geographic and demographic heterogeneities in mobile ownership and the details of phone sharing practices in Africa remain largely unknown [7], [8].

Here, we analyze a randomized survey on mobile phone ownership and usage in Kenya from 2009, originally conducted as a financial survey. We compare the demographics of mobile phone owners, sharers, and non-users, and analyze the geographic and socioeconomic variability among these groups. As expected, poor, rural women are the most under-represented group among mobile phone owners, and phone sharing practices are extremely common in rural areas. This will have important implications both for studies of mobile phone call data records and for mHealth applications in Kenya and elsewhere in Africa.

## Methods

The Financial Sector Deepening Kenya (FSDK) survey asked 32,748 individuals located at 646 communities in 2009 several questions about mobile phone usage, ownership, and monthly expenditure on airtime, as well as detailed demographic questions concerning income, education level and housing type. Cluster stratified probability sampling, based on NASSEP IV (National Sample Survey and Evaluation Program provided by the National Bureau of Statistics) ensured representative populations were included in the survey. First level selection (cluster level) yielded a representative set at the national, provincial, and urbanization levels in each province (see Tables S1 and S2 for details of the survey). The Kenyan National Bureau of Statistics (KNBS) determined how many clusters should be selected for each province, with clusters being randomly selected from a list in the sampling frame for each region to ensure urban regions were adequately represented. Second level selection (household level) of households aimed for ten households within each cluster based on standard sample size calculations. Finally, third level selection (individual level) of individuals aged 16+ years was performed using a standard Kish grid (available in the original survey at http://www.fsdkenya.org). Given the financial literacy goal of the original survey, individuals under 16 were not questioned.

## Results

### Individual patterns of mobile phone ownership and sharing

**Figure 1:**
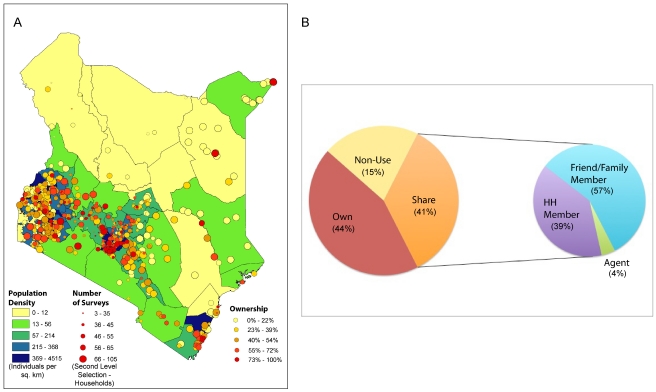
A description of mobile phone ownership and sharing practices in Kenya. A) Mobile phone ownership in Kenya. Map showing the survey locations (based on 2^nd^ level selection) and number of surveys across the country as part of the FSDK 2009. Map background is divided into counties, and colored according to population density (see color bars). B) Proportion of Kenyans who own or use a mobile phone, and proportion of non-owners who share a phone. Of those who share (left), the second pie chart shows who they share with (household (HH) member, friend or family member, or local mobile phone agent.

Figure 1A illustrates the location of each survey, the number of individuals surveyed and the level of mobile phone ownership at each site, as well as the county-level population density. We first aggregated all individuals in the survey to compare the characteristics of mobile phone owners, sharers, and non-users (Table S1). Remarkably, 85% of the individuals surveyed indicated that they used a mobile phone, although only approximately 44% owned their own phone. Figure 1B illustrates the overall proportions of phone owners, sharers, and non-users, as well as the prevalence of phone sharing between family members and friends. Socioeconomic and demographic differences between these groups were pronounced. As expected, mobile phone owners had the highest mean monthly income at 16,400 Kenyan shillings (KSH) (where $1 USD≈75 KSH, 90% range for owners: 2,000–50,000), followed by sharers (6,500 KSH, 90% range: 1,000–20,000), and lastly individuals who did not use a mobile phone (mean: 6,100, 90% range: 1,000–15,000). As a group, phone sharers were mainly female (65%) and spouses of the head of household (60%). The majority of phone sharers used a family member's or friend's phone (57%) followed by another household member's phone (39%). Individuals who did not use a phone at all were also primarily female (81% of this group), married (62%), had no education (40%), and/or were effectively illiterate (62%).

**Figure 2:**
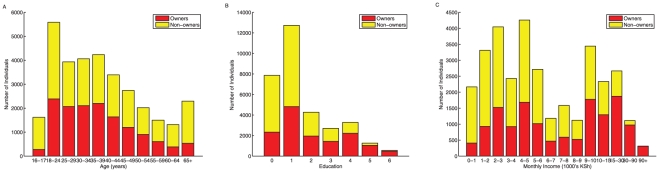
The relationship between mobile phone ownership and socioeconomic attributes. Income (A), age (B), and education (0 = None, 1 = Some primary, 2 = Primary completed, 3 = Some secondary, 4 = Secondary completed, 5 = Technical training, 6 = University), (C) for all individuals in the survey. Binned values reflect the structure of the survey questions.

Strikingly, in every income bracket and demographic group surveyed there was some level of mobile phone ownership. Even individuals in the lowest income bracket (individuals with incomes less than a 1,000 KSH per month) reported 20% ownership. Figure 2 illustrates the relationship between mobile phone ownership and income (Figure 2A), age (Figure 2B), and education (Figure 2C), for example. Both phone owners and phone sharers reported monthly expenditure on mobile phones, and surprisingly both groups spent approximately the same proportion of their income on airtime on average (13% and 10%, respectively). Expenditures were positively correlated with minimum monthly income (owners: R^2^ = 0.363, p<0.0001 and sharers: R^2^ = 0.12, p<0.0001), but the proportion of income spent on airtime decreased non-linearly as income increased (data not shown). Poor individuals therefore spent a disproportionate amount of their income on airtime, highlighting the perceived importance of mobile phones in the lives of individuals across all income brackets in Kenya.

We performed a multilevel logistic regression where the dependent variable was mobile phone ownership and the unit of analysis was the individual. We focused on key socio-demographic attributes including age, gender, education, effective literacy, and monthly income (see Tables 1 and 2). We constructed a fixed effects model using dummy variables for each county to account for the county membership effects between socio-demographic variables:
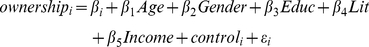
where *β_i_* is the fixed effect for the individual. The coefficients were estimated using ordinary least squares regression (Model AIC: 33318). Education, literacy, and gender were the most important predictors of mobile phone ownership, respectively (see Table 3). We performed additional regressions removing either literacy or education, since these are strongly correlated, but this had little effect on the coefficients (see SI for tables). Age had a small effect on mobile phone ownership since the tails of the age distribution had lower phone ownership. Interestingly, income and education both had little predictive ability to determine mobile phone ownership once the other demographic variables were taken into account (county level differences in distribution were controlled by the fixed effects). See Tables S3 and S4 for more details.

**Table 1:** For the multi-level logistic regression, the variables age, gender, education (educ), literacy (lit), and income were used.

Variable	Description
**Age**	Age of respondent (age range 16–65+)
**Gender**	Gender of respondent (Female (1) or Male (2))
**Educ**	Highest level of education completed (education level between None and University)
**Lit**	Effective literacy level (Illiterate, Mildly Literate, or Literate)
**Income**	Minimum monthly income in 1000 KsH (self-reported minimum amount of KsH necessary to meet basic monthly needs)

A brief description of each variable is provided including the categories used in the survey is shown.

**Table 2:** Correlations between all variables used in the multi-level logistic regression and mobile phone ownership were calculated using a Pearson's product moment correlation test.

	t (degrees of freedom: 32,688)	p-value	Correlation coefficient
**Gender**	19.18	<0.0001	0.105
**Age**	11.23	<0.0001	0.062
**Education Level**	98.65	<0.0001	0.479
**Literacy**	76.34	<0.0001	0.389
**Income**	32.88	<0.0001	0.179

The strongest correlation was between education level and mobile phone ownership.

**Table 3:** A multi-level logistic regression was performed using age, gender, education, literacy, and income to predict mobile phone ownership.

	Estimate	OR	z value	p-value	Std. Error
**Age** 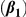	−0.0836	0.92	−15.3420	0.0000	0.0055
**Gender** 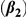	0.2551	1.29	9.0000	0.0000	0.0283
**Education** 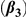	−0.5159	0.60	−40.8650	0.0000	0.0126
**Literacy** 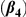	0.4522	1.57	21.4810	0.0000	0.0211
**Income** 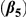	−0.0400	0.96	−21.9030	0.0000	0.0018

The coefficient, odds ratio (OR), standard error, z-value, and p-value for each regressor are shown below. Education was the strongest predictor of mobile phone ownership, whereas income and age had little predictive ability.

### County level patterns of mobile phone ownership

Individual survey results were aggregated to the county level, and compared to data on county-level population density, percent considered urban, and poverty rate as measured in the 2009 National Census. Tables S5 and S6 present baseline statistics for counties with various population estimates and densities (with analyses of counties stratified by percent urban (Table S6) and high and low poverty rate (Table S7)). County-level population density, poverty rate, and percentage of the population considered urban show distinct geographical patterns but are significantly correlated with each other (pairwise correlation coefficient between population density and percentage urban: 0.789, p<0.0001, correlation coefficient between population density and poverty rate: −0.327, p = 0.025, correlation coefficient between percentage urban and poverty rate: −0.345, p = 0.017).

**Figure 3:**
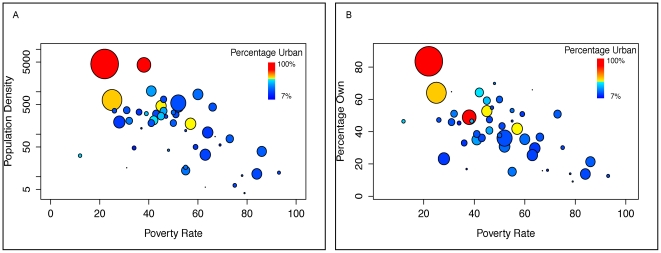
The relationship between population density and poverty rate with mobile phone ownership. A) The relationship between population density and poverty rate in Kenya by county. Each circle represents a county, with the size of the circle corresponding to the total county population, and the color of the circle representing the percent of the population considered urban (see main text). B) The relationship between mobile phone ownership and poverty rate in Kenya by county. Circles sized and colored as above.

**Figure 4:**
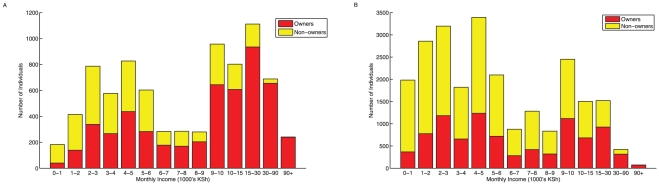
The distribution of mobile phones in different income brackets. Urban A) and rural counties B) are shown separately. Here, urban counties were classified as those having 50% or more of their population considered urban. Rural counties were classified as those having up to 50% of their population considered urban.

As expected, the proportion of individuals owning a mobile phone in each county sample was positively correlated with the population density of the county (R^2^ = 0.35, p = 0.007) and the fraction of the population considered urban (R^2^ = 0.51, p = 0.0001, where an urban area is one with more than 2,000 individuals per 10 km^2^), and negatively correlated with the poverty rate (correlation coefficient = −0.567, p<0.0001, where poverty rate is the proportion of individuals living below the poverty line). Since the population measures are correlated with each other (Figure 3A), these relationships are as expected and follow a similar pattern (Figure 3B). For example, only 9% of individuals surveyed in Marsabit owned a mobile phone, a district in the poor, relatively unpopulated northern region, as opposed to 84% of individuals in Nairobi, the country's capital. In rural areas, mean phone ownership was 39% (90% range: 14%–43%) compared to urban regions where it was 58% (90% range: 65%–80%). To assess the implications of these discrepancies, we analyzed the distribution of mobile phones in different income brackets in rural and urban counties (Figure 4). Interestingly, although proportional ownership was equivalent among the lowest and highest income brackets in both rural and urban counties, ownership increased linearly with income in the urban but not the rural counties (see Figure S1).

**Figure 5:**
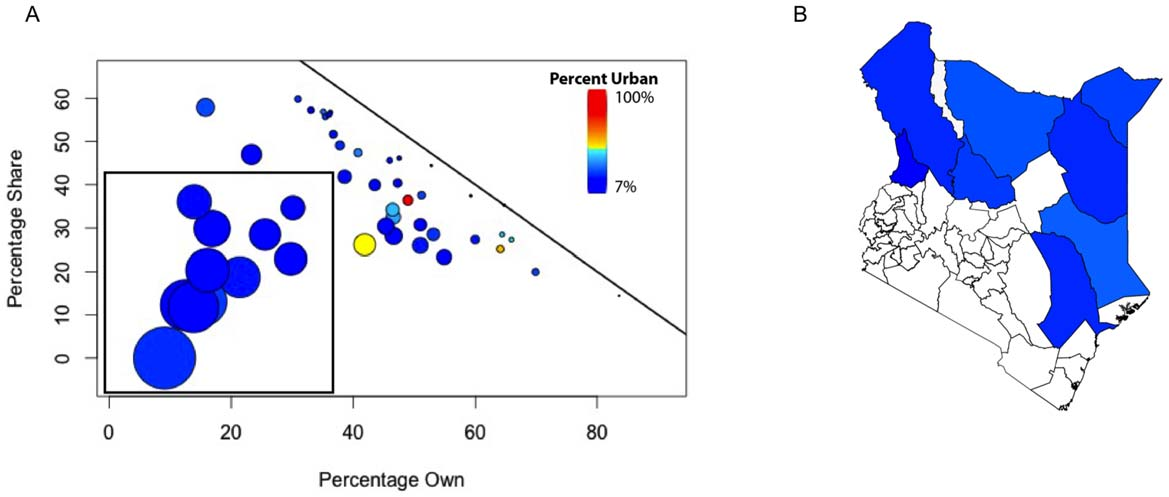
The relationship between mobile phone ownership and sharing. A) The relationship between mobile phone ownership and sharing behavior, by county. Circles are colored by percentage of the population of the country considered urban (see main text), and their size represents the percentage of individuals in a county that doesn't use a mobile phone at all. B) Map showing the counties where less than 30% of individuals own and less than 30% of individuals share a phone. The colors correspond to 3A, representing the percentage of the population considered urban.

There was a strong nonlinear relationship between phone ownership and phone sharing behavior across counties (Figure 5). For most counties, and for all of those with large urban populations, mobile phone ownership and phone sharing were strongly negatively correlated, with the percentage of sharers decreasing as the percentage of owners increases. Counties in the rural northern and eastern parts of Kenya that had a low percentage of owners and sharers, however, exhibited the opposite pattern (see the data points in the box outlined in Figure 5). In these regions where phone ownership was extremely low, phone sharing increased with ownership. Certain communities in very rural areas are therefore in a transition period during which additional mobile phones will be shared by many individuals. Once ownership reaches a certain threshold, however, additional mobile phones decrease the need for sharing. These patterns must be taken into account by studies of behavior based on mobile phone call data records as well as in the design of mHealth applications, since the assumption that each mobile phone or SIM card represents a single individual may not be valid in rural African populations.

## Discussion

Mobile phones offer exciting new ways to engage with and study populations that have traditionally been hard to reach, particularly in the developing world. It is clear that mobile phone ownership and usage is not uniform across populations, however, and that socio-demographic characteristics of owners are not representative of the general population. The heterogeneities in mobile phone ownership described here have important implications for two types of public health application; the analysis of population-level behavior produced passively by mobile phone use, for example in understanding human mobility and the spread of infectious diseases [4], [6], [9], and in mHealth approaches to specific interventions and quality care [10].

Heterogeneous ownership may skew estimates of population dynamics and social networks in Africa. In urban or semi-urban areas this is because we are not capturing data from the least educated individuals, and in rural areas relatively few people have phones at all and phone sharing practices are pervasive. Patterns of phone sharing described here are likely to be found across the developing world; in very isolated areas phone sharing is extremely common and even increases initially as phones penetrate into the community, but as ownership saturates the need for sharing decreases as ownership rises. This phenomenon can complicate analyses that rely on the assumption that each SIM card corresponds to a single individual. Critically, however, every region, income and demographic bracket analyzed here had some level of mobile phone ownership. This suggests that although behavioral measures from mobile phones may be skewed, they will not miss entire sections of society and estimate adjustments may be possible. Furthermore, the penetration of mobile phones is only likely to increase in coming years, which will presumably reduce some of the heterogeneities we have observed.

mHealth approaches targeting remote, hard-to-reach populations where health disparities remain high are becoming increasingly possible as mobile phones become cheaper and more accessible in the developing world [11]. For example, programs to improve insecticide-treated net (ITN) use, compliance to antivirals, and public health messaging for cholera have been employed in several countries [12], [13], [14], [15], [16], [17]. These programs hinge on being able to reach at-risk individuals and on the literacy of the target audience, however. In the FSDK survey, 62% of individuals who did not own a phone were effectively illiterate. Even if mobile phones reached this group, or were supplied to them by particular programs, text-based interventions would not be effective. Furthermore, the gender and socioeconomic heterogeneities inherent in ownership and usage patterns, with poor rural women being significantly under-represented, suggest that maternal health programs may struggle to engage with the highest risk individuals. Our data also suggest that in rural areas, programs that supply phones for longitudinal or individual engagement are likely to be used by multiple people. These programs may fare better in urban centers, however. Similarly, programs targeting populations at risk for drought or famine are likely to have trouble reaching areas most affected, since rural populations and farming communities tend to have low mobile phone ownership. Taking regional differences in mobile phone ownership into account is critical, therefore, if mHealth approaches are to be effective.

## Supporting Information

Figure S1 **Normalized Percentage Owners and Non-Owners for Each Income Bracket in Rural Counties and Urban Counties.**
(EPS)

Table S1 The differences in socio-demographic characteristics between owners, sharers, and non-users. For each category, the percentage of owners, non-owners who share and difference between groups is shown. For categorical variables, a chi-squared test was used to quantify the difference between the groups. For the continuous variables, an ANOVA was used.
(DOCX)

Table S2 Number of Counties and Individual Surveys in Each County Level Category.
(DOCX)

Table S3 Correlations between variables used in the regression analysis.
(DOCX)

Table S4 Coefficient results when either education or literacy was omitted from the multilevel regression.
(DOCX)

Table S5 Overview of mean percentage of individuals surveyed in each category. 5^th^ and 95^th^ quantile values are show in parentheses. Individual surveys were aggregated to their county location based on the location of the household. Counties were then aggregated by population density (high and low) with Nairobi kept separate. Low population density counties have below the mean population density per county (less than 408 individuals per square kilometer). High population density counties have equal to or above the mean population density per county.
(DOCX)

Table S6 Mean Percentage of Respondents in Each Category Per County By Percentage Rural. County level values were aggregated based on rural (percentage of the population rural greater than 50%) or urban (percentage of the population urban greater than 50%). The capital, Nairobi, was not aggregated with other counties. 5^th^ and 95^th^ quantile values are shown in parentheses.
(DOCX)

Table S7 Mean Percentage of Respondents in Each Category Per County by Poverty Rate. County level values were aggregated based on poverty rate with high poverty rate counties (poverty rate greater than 50%) or low poverty rate counties (poverty rate less than 50%). The capital, Nairobi, was not aggregated with other counties. 5^th^ and 95^th^ quantile values are shown in parentheses.
(DOCX)
