# Supplementary material for: Heterogeneous Mobile Phone Ownership and Usage Patterns in Kenya
Source: PLoS One. 2012 Apr 25;7(4):e35319. doi: 10.1371/journal.pone.0035319 (PMC3338828; doi:10.1371/journal.pone.0035319)
Supplement: Table S1 — The differences in socio-demographic characteristics between owners, sharers, and non-users. For each category, the percentage of owners, non-owners who share and difference between groups is shown. For categorical variables, a chi-squared test was used to quantify the difference between the groups. For the continuous variables, an ANOVA was used. (DOCX) [file pone.0035319.s002.docx]

**Table 1: The differences in socio-demographic characteristics between owners, sharers, and non-users.** For each category, the percentage of owners, non-owners who share and difference between groups is shown. For categorical variables, a chi-squared test was used to quantify the difference between the groups. For the continuous variables, an ANOVA was used.

| **Category** | **Ownership (%)** | **Non-owners who share (%)** | **Statistical test for difference** | **Adjusted** **P-value** |
| --- | --- | --- | --- | --- |
| **Gender** |  |  |  |  |
| Male | 45 | 45 | X^2^ = 364.23 | <0.0001 |
| Female | 37 | 60 |  |  |
| **Education** |  |  | X^2^ = 4021.88 | <0.0001 |
| None | 14 | 31 |  |  |
| Some Primary | 29 | 48 |  |  |
| Primary Completed | 45 | 40 |  |  |
| Some Secondary | 53 | 40 |  |  |
| Secondary Completed | 77 | 20 |  |  |
| Technical Training | 95 | 4 |  |  |
| University | 83 | 7 |  |  |
| **Occupation** |  |  | X^2^=2738.45 | <0.0001 |
| Farm/Domestic worker | 29 | 71 |  |  |
| Pensioner/Dependent | 38 | 62 |  |  |
| Fisherman | 39 | 61 |  |  |
| Farm owner | 48 | 52 |  |  |
| Business owner | 56 | 44 |  |  |
| Land/Property Owner | 61 | 39 |  |  |
| Private sector | 80 | 20 |  |  |
| Government | 96 | 4 |  |  |
| **Roof type** |  |  | X^2^ = 1825.13 | <0.0001 |
| Concrete | 92 | 8 |  |  |
| Abestos sheets | 92 | 7 |  |  |
| Tiles | 88 | 10 |  |  |
| Iron | 48 | 37 |  |  |
| Tin | 45 | 5 |  |  |
| Grass | 25 | 43 |  |  |
| Makuti | 13 | 33 |  |  |
| **Literate** |  |  | X^2^ = 2381.47 | <0.0001 |
| No | 15 | 40 |  |  |
| Functionally | 30 | 42 |  |  |
| Yes | 58 | 32 |  |  |
| **Age** |  |  |  | 0.018 |
| 16-17 | 17 | 51 | F = 5.61 |  |
| 18-24 | 43 | 41 |  |  |
| 25-29 | 53 | 33 |  |  |
| 30-34 | 52 | 31 |  |  |
| 35-39 | 52 | 30 |  |  |
| 40-44 | 48 | 34 |  |  |
| 45-49 | 44 | 31 |  |  |
| 50-54 | 45 | 33 |  |  |
| 55-59 | 41 | 31 |  |  |
| 60-64 | 29 | 42 |  |  |
| 65+ | 23 | 39 |  |  |
| **Income (1000 Ksh)** |  |  | F = 665.43 | <0.0001 |
| 0-1 | 19 | 49 |  |  |
| 1-5 | 36 | 42 |  |  |
| 5-10 | 44 | 31 |  |  |
| 10-30 | 59 | 29 |  |  |
| 30-60 | 83 | 13 |  |  |
| 60-90 | 90 | 4 |  |  |
| 90+ | 98 | 0.6 |  |  |
