# Supplementary material for: Heterogeneous Mobile Phone Ownership and Usage Patterns in Kenya
Source: PLoS One. 2012 Apr 25;7(4):e35319. doi: 10.1371/journal.pone.0035319 (PMC3338828; doi:10.1371/journal.pone.0035319)
Supplement: Table S2 — Number of Counties and Individual Surveys in Each County Level Category. (DOCX) [file pone.0035319.s003.docx]

**Table S2: Number of Counties and Individual Surveys in Each County Level Category.**

|  | Rural | Urban | Low  Pop. Density | High  Pop. Density | High  Poverty Rate | Low  Poverty Rate | Nairobi |
| --- | --- | --- | --- | --- | --- | --- | --- |
| Number  of  counties | 42 | 4 | 37 | 9 | 22 | 24 | 1 |
| Number  of  individual surveys | 15,651 | 5,318 | 19,564 | 10,069 | 13,982 | 15,651 | 2722 |
