# Supplementary material for: Heterogeneous Mobile Phone Ownership and Usage Patterns in Kenya
Source: PLoS One. 2012 Apr 25;7(4):e35319. doi: 10.1371/journal.pone.0035319 (PMC3338828; doi:10.1371/journal.pone.0035319)
Supplement: Table S3 — Correlations between variables used in the regression analysis. (DOCX) [file pone.0035319.s004.docx]

**Table S2: Correlations between variables used in the regression analysis.**

|  | Gender | Age | Education | Literacy | Income |
| --- | --- | --- | --- | --- | --- |
| Gender | 1 | 0.125 | 0.099 | 0.106 | 0.0228 |
| Age |  | 1 | -0.242 | -0.331 | -0.0131 |
| Education |  |  | 1 | 0.638 | 0.243 |
| Literacy |  |  |  | 1 | 0.12 |
| Income |  |  |  |  | 1 |
