# Supplementary material for: Heterogeneous Mobile Phone Ownership and Usage Patterns in Kenya
Source: PLoS One. 2012 Apr 25;7(4):e35319. doi: 10.1371/journal.pone.0035319 (PMC3338828; doi:10.1371/journal.pone.0035319)
Supplement: Table S4 — Coefficient results when either education or literacy was omitted from the multilevel regression. (DOCX) [file pone.0035319.s005.docx]

**Table S3: Coefficient results when either education or literacy was omitted from the multilevel regression.**

|  | Coeff. Omit Educ | OR Omit Educ | Coeff. Omit Lit | OR Omit Lit |
| --- | --- | --- | --- | --- |
| Gender | 0.337 | 1.40 | 0.318 | 1.37 |
| Age | 0.055 | 1.06 | 0.051 | 1.05 |
| Education | OMIT | OMIT | 0.647 | 1.91 |
| Literacy | 0.860 | 2.36 | OMIT | OMIT |
| Income | -0.054 | 0.95 | -0.043 | 0.96 |
