# Supplementary material for: Heterogeneous Mobile Phone Ownership and Usage Patterns in Kenya
Source: PLoS One. 2012 Apr 25;7(4):e35319. doi: 10.1371/journal.pone.0035319 (PMC3338828; doi:10.1371/journal.pone.0035319)
Supplement: Table S5 — Overview of mean percentage of individuals surveyed in each category. 5th and 95th quantile values are show in parentheses. Individual surveys were aggregated to their county location based on the location of the household. Counties were then aggregated by population density (high and low) with Nairobi kept separate. Low population density counties have below the mean population density per county (less than 408 individuals per square kilometer). High population density counties have equal to or above the mean population density per county. (DOCX) [file pone.0035319.s006.docx]

**Table S5: Overview of mean percentage of individuals surveyed in each category.** 5^th^ and 95^th^ quantile values are show in parentheses. Individual surveys were aggregated to their county location based on the location of the household. Counties were then aggregated by population density (high and low) with Nairobi kept separate. Low population density counties have below the mean population density per county (less than 408 individuals per square kilometer). High population density counties have equal to or above the mean population density per county.

|  | Low Population Density | High Population Density | Nairobi |
| --- | --- | --- | --- |
| Gender |  |  |  |
| Male | 50% (47, 54) | 49% (48, 52) | 49% |
| Female | 50% (46, 53) | 51% (48, 52) | 51% |
| Education |  |  |  |
| None | 29% (12, 66) | 20% (13, 26) | 14% |
| Some Primary | 39% (26, 51) | 45% (34, 54) | 20% |
| Primary Complete | 13% (4, 20) | 12% (8, 15) | 11% |
| Some Secondary | 7% (2, 12) | 9% (6, 13) | 11% |
| Secondary Complete | 8% (1, 15) | 10% (5, 16) | 22% |
| Technical Training | 3% (0, 7) | 3% (1, 6) | 14% |
| University | 1% (0, 3) | 1% (1, 3) | 10% |
| Age |  |  |  |
| 16-17 | 6% (2, 14) | 5% (2, 7) | 5% |
| 18-24 | 7% (2, 19) | 4% (3, 5) | 1% |
| 25-29 | 9% (1, 20) | 8% (6, 11) | 3% |
| 30-34 | 15% (8, 24) | 17% (13, 23) | 30% |
| 35-39 | 12% (6, 23) | 11% (8, 15) | 20% |
| 40-44 | 13% (7, 23) | 13% (8, 17) | 13% |
| 45-49 | 13% (6, 19) | 12% (9, 13) | 10% |
| 50-54 | 10% (4, 15) | 12% (9, 15) | 7% |
| 55-59 | 8% (2, 16) | 8% (4, 11) | 6% |
| 60-64 | 6% (2, 10) | 6% (3, 8) | 3% |
| 65+ | 5% (1, 11) | 5% (4, 8) | 2% |
| Literacy |  |  |  |
| Literate | 60% (22, 84) | 68% (59, 79) | 94% |
| Mildly Literate | 8% (1, 20) | 4% (1, 8) | 3% |
| Illiterate | 35% (13, 71) | 28% (17, 36) | 3% |
| Roof Type |  |  |  |
| Corr. Iron Sheet | 70% (18, 99) | 82% (61, 95) | 54% |
| Tiles | 17% (0, 72) | 4% (1, 11) | 24% |
| Concrete | 15% (0, 39) | 9% (2, 31) | 14% |
| Asbestos Sheets | 4% (1, 10) | 7% (2, 17) | 9% |
| Grass | 17% (2, 37) | 3% (1, 6) | 0% |
| Maktui | 0% (0, 0) | 15% (2, 29) | 0% |
| Tin | 0% (0, 0) | 0% (0, 0) | 0% |
| Other | 0% (0, 0) | 0% (0, 0) | 0% |
| Income |  |  |  |
| 0--1 | 8% (0, 23) | 9% (2, 17) | 1% |
| 1--5 | 43% (18, 65) | 53% (36, 64) | 14% |
| 5--10 | 33% (11, 60) | 23% (14, 34) | 28% |
| 10--15 | 8% (1, 17) | 5% (2, 10) | 13% |
| 15--90 | 9% (1, 25) | 9% (2, 19) | 37% |
| 90+ | 0% (0, 2) | 0% (0, 1) | 8% |
