# Supplementary material for: Heterogeneous Mobile Phone Ownership and Usage Patterns in Kenya
Source: PLoS One. 2012 Apr 25;7(4):e35319. doi: 10.1371/journal.pone.0035319 (PMC3338828; doi:10.1371/journal.pone.0035319)
Supplement: Table S6 — Mean Percentage of Respondents in Each Category Per County By Percentage Rural. County level values were aggregated based on rural (percentage of the population rural greater than 50%) or urban (percentage of the population urban greater than 50%). The capital, Nairobi, was not aggregated with other counties. 5th and 95th quantile values are shown in parentheses. (DOCX) [file pone.0035319.s007.docx]

**Table S6: Mean Percentage of Respondents in Each Category Per County By Percentage Rural.** County level values were aggregated based on rural (percentage of the population rural greater than 50%) or urban (percentage of the population urban greater than 50%). The capital, Nairobi, was not aggregated with other counties. 5^th^ and 95^th^ quantile values are shown in parentheses.

|  | Rural | Urban | Nairobi |
| --- | --- | --- | --- |
| Gender |  |  |  |
| Male | 50% (47, 53) | 50% (49, 52) | 49% |
| Female | 50% (47, 53) | 50% (48, 51) | 51% |
| Education |  |  |  |
| None | 20% (12, 41) | 18% (12, 26) | 14% |
| Some Primary | 40% (30, 50) | 38% (34, 43) | 20% |
| Primary Complete | 15% (8, 21) | 15% (13, 18) | 11% |
| Some Secondary | 9% (5, 13) | 10% (7, 13) | 11% |
| Secondary Complete | 11% (6, 17) | 13% (11, 16) | 22% |
| Technical Training | 4% (1, 7) | 5% (2, 6) | 14% |
| University | 1% (1, 3) | 2% (1, 3) | 10% |
| Age |  |  |  |
| 16-17 | 5% (2, 8) | 4% (2, 6) | 5% |
| 18-24 | 6% (3, 18) | 5% (4, 5) | 1% |
| 25-29 | 10% (4, 19) | 7% (6, 9) | 3% |
| 30-34 | 17% (9, 25) | 17% (9, 24) | 30% |
| 35-39 | 13% (7, 19) | 12% (6, 16) | 20% |
| 40-44 | 12% (6, 16) | 11% (10, 12) | 13% |
| 45-49 | 13% (6, 19) | 12% (11, 13) | 10% |
| 50-54 | 9% (4, 13) | 13% (10, 17) | 7% |
| 55-59 | 8% (2, 18) | 9% (4, 14) | 6% |
| 60-64 | 5% (1, 9) | 6% (3, 9) | 3% |
| 65+ | 5% (1, 11) | 5% (4, 7) | 2% |
| Literacy |  |  |  |
| Literate | 69% (49, 84) | 74% (69, 81) | 94% |
| Mildly Literate | 8% (1, 21) | 5% (1, 10) | 3% |
| Illiterate | 25% (13, 38) | 21% (15, 28) | 3% |
| Roof Type |  |  |  |
| Corr. Iron Sheet | 85% (56, 99) | 83% (65, 95) | 54% |
| Tiles | 4% (0, 11) | 3% (1, 4) | 24% |
| Concrete | 9% (0, 29) | 2% (1, 3) | 14% |
| Asbestos Sheets | 3% (1, 10) | 3% (2, 4) | 9% |
| Grass | 6% (1, 18) | 3% (1, 6) | 0% |
| Maktui | 15% (2, 29) | 15% (2, 29) | 0% |
| Tin | 0% (0, 0) | 0% (0, 0) | 0% |
| Other | 0% (0, 0) | 0% (0, 0) | 0% |
| Income (thousand KSH) |  |  |  |
| 0 – 1 | 9% (0, 21) | 3% (2, 5) | 1% |
| 1 – 5 | 45% (19, 65) | 44% (36, 57) | 14% |
| 5 – 10 | 31% (12, 60) | 31% (27, 34) | 28% |
| 10 – 15 | 7% (2, 16) | 8% (5, 11) | 13% |
| 15 – 90 | 9% (1, 25) | 14% (6, 19) | 37% |
| 90 + | 0% (0, 1) | 0% (0, 1) | 8% |
