# Supplementary material for: Heterogeneous Mobile Phone Ownership and Usage Patterns in Kenya
Source: PLoS One. 2012 Apr 25;7(4):e35319. doi: 10.1371/journal.pone.0035319 (PMC3338828; doi:10.1371/journal.pone.0035319)
Supplement: Table S7 — Mean Percentage of Respondents in Each Category Per County by Poverty Rate. County level values were aggregated based on poverty rate with high poverty rate counties (poverty rate greater than 50%) or low poverty rate counties (poverty rate less than 50%). The capital, Nairobi, was not aggregated with other counties. 5th and 95th quantile values are shown in parentheses. (DOCX) [file pone.0035319.s008.docx]

**Table S7: Mean Percentage of Respondents in Each Category Per County by Poverty Rate.** County level values were aggregated based on poverty rate with high poverty rate counties (poverty rate greater than 50%) or low poverty rate counties (poverty rate less than 50%). The capital, Nairobi, was not aggregated with other counties. 5^th^ and 95^th^ quantile values are shown in parentheses.

|  | High Poverty Rate | Low Poverty Rate | Nairobi |
| --- | --- | --- | --- |
| Gender |  |  |  |
| Male | 50% (48, 54) | 50% (47, 53) | 49% |
| Female | 50% (46, 52) | 50% (47, 53) | 51% |
| Education |  |  |  |
| None | 35% (14, 66) | 20% (12, 41) | 14% |
| Some Primary | 40% (25, 55) | 40% (30, 50) | 20% |
| Primary Complete | 10% (4, 18) | 15% (8, 21) | 11% |
| Some Secondary | 6% (1, 11) | 9% (5, 13) | 11% |
| Secondary Complete | 6% (1, 11) | 11% (6, 17) | 22% |
| Technical Training | 2% (0, 4) | 4% (1, 7) | 14% |
| University | 1% (0, 1) | 1% (1, 3) | 10% |
| Age |  |  |  |
| 16-17 | 7% (2, 15) | 5% (2, 8) | 5% |
| 18-24 | 6% (2, 18) | 6% (3, 18) | 1% |
| 25-29 | 8% (1, 17) | 10% (4, 19) | 3% |
| 30-34 | 14% (7, 21) | 17% (9, 25) | 30% |
| 35-39 | 11% (4, 21) | 13% (7, 19) | 20% |
| 40-44 | 15% (7, 24) | 12% (6, 16) | 13% |
| 45-49 | 12% (6, 19) | 13% (6, 19) | 10% |
| 50-54 | 11% (7, 17) | 9% (4, 13) | 7% |
| 55-59 | 8% (2, 13) | 8% (2, 18) | 6% |
| 60-64 | 7% (3, 11) | 5% (1, 9) | 3% |
| 65+ | 5% (2, 8) | 5% (1, 11) | 2% |
| Literacy |  |  |  |
| Literate | 53% (17, 80) | 69% (49, 84) | 94% |
| Mildly Literate | 7% (1, 15) | 8% (1, 21) | 3% |
| Illiterate | 44% (21, 72) | 25% (13, 38) | 3% |
| Roof Type |  |  |  |
| Corr. Iron Sheet | 58% (16, 95) | 85% (56, 99) | 54% |
| Tiles | 27% (0, 80) | 4% (0, 11) | 24% |
| Concrete | 23% (2, 55) | 9% (0, 29) | 14% |
| Asbestos Sheets | 7% (1, 16) | 3% (1, 10) | 9% |
| Grass | 40% (40, 40) | 6% (1, 18) | 0% |
| Maktui | 0% (0, 0) | 15% (2, 29) | 0% |
| Tin | 0% (0, 0) | 0% (0, 0) | 0% |
| Other | 0% (0, 0) | 0% (0, 0) | 0% |
| Income |  |  |  |
| 0--1 | 10% (0, 30) | 6% (2, 11) | 1% |
| 1--5 | 44% (14, 65) | 45% (27, 63) | 14% |
| 5--10 | 30% (9, 59) | 31% (17, 58) | 28% |
| 10--15 | 8% (0, 22) | 7% (2, 11) | 13% |
| 15--90 | 8% (1, 15) | 10% (1, 24) | 37% |
| 90+ | 0% (0, 1) | 1% (0, 3) | 8% |
